# Against All Odds: Why a Lung Donor Score Does Not Add Up

**DOI:** 10.3389/ti.2025.14937

**Published:** 2025-10-17

**Authors:** Simon Schwab, Fabian Iten

**Affiliations:** ^1^ Swisstransplant, Bern, Switzerland; ^2^ Center for Reproducible Science and Research Synthesis, University of Zurich, Zurich, Switzerland; ^3^ Faculty of Medicine, University of Bern, Bern, Switzerland

**Keywords:** lung transplant, risk factors, clinical prediction model, lung donor score, statistical modeling

Dear Editors,

The Eurotransplant Lung Donor Score (LDS) [1] is a prediction model to assess the risk of donor lung discard due to medical reasons. A logistic regression model was fitted using data from over 5,000 donor lungs, and the resulting odds ratios were translated into a risk score ranging from 6 to 19 points, with higher scores indicating poorer donor lung quality. The LDS was found to be significantly associated with 1-year survival. The authors concluded that the LDS accurately reflects the likelihood of organ acceptance and is predictive of patient mortality. Furthermore, its implementation at the time of donor reporting may support more informed donor risk assessment and improve recipient selection. The LDS is used to study the quality of transplanted lungs in Eurotransplant [2], but it’s unclear to what extent it is actually used in clinical practice to define an extended-criteria donor lung.

However, we believe the Eurotransplant LDS has some methodological limitations. The scoring system is presented in Table 2 of the original report. Odds ratios from the logistic regression model were rounded to the nearest whole number to construct a risk score. For example, donor age between 45 and 54 years was assigned 1 point (odds ratio 1.33), a history of smoking was assigned 2 points (odds ratio 1.53), and the presence of inflammation on donor bronchoscopy was assigned 3 points (odds ratio 2.83). Finally, individual points were summed into an overall risk score. We identified four limitations in the construction of the LDS, discussed in detail below.

First, rounding odds ratios such as 1.53, 1.87, and 2.40 all to a score of 2 points is suboptimal, as it obscures important differences in risk. These values represent varying levels of association with organ discard: a donor smoking history corresponds to a 1.5-fold increase in the odds of organ discard, purulent secretions observed on bronchoscopy to a 1.9-fold increase, and a PaO_2_/FiO_2_ ratio of 301–350 mmHg to a 2.4-fold increase. Grouping them under the same score point may discard a vast amount of information, which may harm discrimination and the predictive accuracy of the model.

Second, the construction relies on adding odds ratios, a practice that is statistically not valid. For example, consider a 20-year-old donor with purulent secretions observed on bronchoscopy. The donor is assigned the level of donor age <45, resulting in an odds ratio of 1 (reference level), indicating neither a decrease nor an increase in the odds of organ discard. Then, the odds ratio for purulent secretions is nearly 2, reflecting a 2-fold increased chance of discard. Combining these two odds ratios would result in a 1 × 2 = 2-fold increase in the odds of organ discard. If these two odds ratios were combined by summing them, it would result in a 3-fold increase in the odds of organ discard, which is incorrect. Odds ratios multiply; they do not add up. This is a basic mathematical property of ratios.

Third, caution is warranted when using binary indicators to denote the presence or absence of missing data. In the current model, nearly all donor risk factors include a category specifically representing missing values, which may introduce bias and compromise the validity of the predictions [3]. A single imputation model may be considered when the missing data are low. Advanced methods, such as multiple imputations, are not always practical when applying a model in clinical practice. Predictors with substantial missingness are generally poor candidates, as they are also likely to be missing in future patients. When only a small proportion (less than 5%) of data is missing, complete case analysis may be acceptable, though it remains important to always investigate potential reasons for missingness. Strong candidate predictors are objective measures that are consistently and widely available. In organ transplantation, in particular, key clinical information is mandatory, as an organ cannot be allocated without it. Thus, it is often possible to retrieve missing data from the medical records and databases.

Fourth, the primary outcome used in the model was organ discard. As it stands, the model predicts the probability of discard rather than clinically meaningful endpoints such as graft failure or patient death. Even if organ discard is correlated with these outcomes, the primary endpoint should ideally reflect a patient-centered outcome following transplantation.

We propose the following simple solution: we calculated the log odds ratios from Table 2 of the original publication. We then multiplied these values by a factor of 4 and rounded them to whole numbers. This preserved sufficient accuracy while aligning more closely with the original score for easier comparison. Using a multiplicative factor of 5 would also work, but it would increase the maximal score to 30. This new risk score system avoids data loss due to rounding of small numbers. Moreover, adding the values on the log scale is statistically appropriate. The new risk score ranges from 0 to 24, and is shown in Table 1. Supplementary Figure 1A shows the distribution of the original and revised scores, while Supplementary Figure 1B compares them directly. The revised score, however, cannot address the issues with how missing data were handled and with the choice of the primary outcome.

**TABLE 1:** Our revised score along with the original results by Eurotransplant (we preserved the ordering of the risk variables). The revised score is the log OR multiplied by a factor of 4 and rounded to a whole number. For example, a visualized tumor in the bronchoscopy gets 4 × 1.68 ∼ 7 points.

Factor	OR	log OR	Original score	Revised score
Donor age (y)				
<45	1.00	0.00	1	0
45–54	1.33	0.29	1	1
55–59	1.77	0.57	2	2
60+	2.68	0.99	3	4
Donor history				
compromised	3.90	1.36	4	5
uncompromised	1.00	0.00	1	0
Smoking history				
yes	1.53	0.43	2	2
no	1.00	0.00	1	0
missing	1.18	0.17	1	1
Chest X-ray				
clear	1.00	0.00	1	0
edema	1.28	0.25	1	1
shadow	1.65	0.50	2	2
atelectasis	1.31	0.27	1	1
consolidation	1.58	0.46	2	2
missing	1.23	0.21	1	1
Bronchoscopy				
clear	1.00	0.00	1	0
nonpurulent	1.48	0.39	1	2
purulent	1.87	0.63	2	3
inflammation	2.83	1.04	3	4
visualized tumor	5.34	1.68	5	7
missing	1.26	0.23	1	1
PO2/FiO2 (mmHg)				
>450	1.00	0.00	1	0
351–450	1.26	0.23	1	1
301–350	2.40	0.88	2	4
<=300	2.97	1.09	3	4
missing	2.35	0.85	2	3
		Range	6–19	0–24

Medical statisticians warned against the flawed practice of adding up ratios decades ago [4] but the problem seems to persist. A risk score for donation after cardiac death (DCD) liver transplants combined hazard ratios in a similarly incorrect manner [5]. Summing odds ratios (or hazard ratios) not only lacks any meaningful interpretation, but may also produce misleading results. For instance, an odds ratio of 0.8 from a logistic model, which indicates a 0.8-fold risk reduction, would paradoxically increase the overall risk score if added. On the log scale, however, ratios smaller than 1 have a negative sign.

The LDS continues to be used today, for instance, a recent study used it as a benchmark to compare with their newly developed risk score [6]. Had the LDS been constructed using a statistically appropriate approach, the resulting score may have offered more accurate and evidence-based weighting of clinical donor risk factors in lung transplants. We hope our approach offers a constructive refinement of the LDS that could improve its accuracy and interpretability.

## Data Availability

The original contributions presented in the study are included in the article/supplementary material, further inquiries can be directed to the corresponding author.

## Supplementary Material

The SupplementaryMaterial for this article can be found online at: https://www.frontierspartnerships.org/articles/10.3389/ti.2025.14937/full#supplementary-material

Supplementary Figure 1
**(A)** Histogram showing the two distributions, and **(B)** scatterplot comparing the original Lung Donor Score (LDS) to our revised score. Donors assigned 6, 7, 8, etc. points in the original model receive more nuanced values in the revised version; this applies across the entire risk spectrum. Data were based on simulations of 10,000 lung donors, representing all theoretically possible combinations of risk factors. The simulations do not accurately reflect real-world distributions and covariances among risk variables, but may be suitable for the purpose of visualizing our argument that a significant amount of information was discarded when constructing the LDS.
