# Supplementary figures and images for: Against All Odds: Why a Lung Donor Score Does Not Add Up
Source: Transpl Int. 2025 Oct 17;38:14937. doi: 10.3389/ti.2025.14937 (PMC12590763; doi:10.3389/ti.2025.14937)

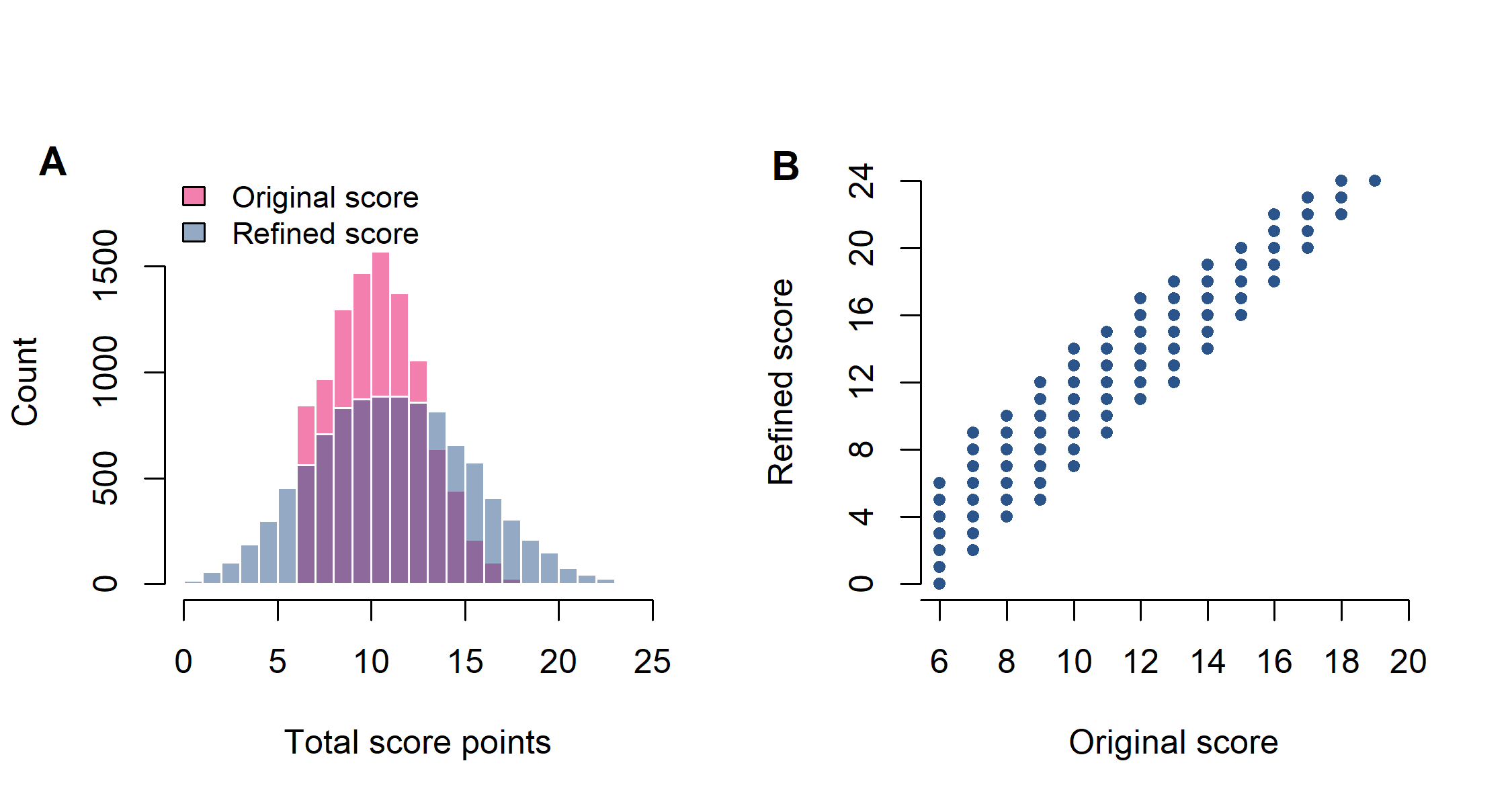

Supplement: Supplementary file 1 [file Image1.tiff]
